# Feasibility and Acceptability of a Remotely Delivered, Web-Based Behavioral Intervention for Men With Prostate Cancer: Four-Arm Randomized Controlled Pilot Trial

**DOI:** 10.2196/19238

**Published:** 2020-12-31

**Authors:** June M Chan, Erin L Van Blarigan, Crystal S Langlais, Shoujun Zhao, Justin W Ramsdill, Kimi Daniel, Greta Macaire, Elizabeth Wang, Kellie Paich, Elizabeth R Kessler, Tomasz M Beer, Karen S Lyons, Jeanette M Broering, Peter R Carroll, Stacey A Kenfield, Kerri M Winters-Stone

**Affiliations:** 1 University of California, San Francisco San Francisco, CA United States; 2 Oregon Health and Science University Portland, OR United States; 3 Vagelos College of Physicians and Surgeons Columbia University New York, NY United States; 4 Movember Foundation Culver City, CA United States; 5 University of Colorado Aurora, CO United States; 6 Boston College Boston, MA United States

**Keywords:** diet, physical activity, exercise, lifestyle, cancer, survivorship, text messages, internet

## Abstract

**Background:**

Diet and exercise may be associated with quality of life and survival in men with prostate cancer.

**Objective:**

This study aimed to determine the feasibility and acceptability of a remotely delivered web-based behavioral intervention among men with prostate cancer.

**Methods:**

We conducted a multi-site 4-arm pilot randomized controlled trial of a 3-month intervention (TrueNTH Community of Wellness). Eligibility included self-reported prostate cancer diagnosis, having a personal device that connected to the internet, age ≥18 years, and ability to read English and receive text messages and emails. Men receiving chemotherapy or radiation, or those who reported contraindications to exercise, could participate with physician clearance. Participants were randomized (1:1:1:1) to additive intervention levels: website; website and personalized diet and exercise prescription; website, personalized prescription, Fitbit, and text messages; and website, personalized prescription, Fitbit, text messages, and 2 30-minute phone calls—one with an exercise trainer and one with a registered dietician. Primary outcomes were feasibility (accrual and attrition) and acceptability (survey data and website use). We described self-reported diet and exercise behavior at the time of enrollment, 3 months, and 6 months as secondary outcomes.

**Results:**

In total, 202 men consented and were randomized between August 2017 and September 2018 (level 1: 49, level 2: 51, level 3: 50, level 4: 52). A total of 160 men completed the onboarding process and were exposed to their randomly assigned intervention (38, 38, 42, and 42 in levels 1, 2, 3, and 4, respectively). The follow-up rate was 82.7% (167/202) at 3 months and 77.2% (156/202) at 6 months. Participants had a median age of 70 years and were primarily White and college educated. Website visit frequency over the 3-month intervention period increased across levels (median: 2, 9, 11, and 16 visits for levels 1, 2, 3, and 4, respectively). Most were satisfied or very satisfied with the intervention (20/39, 51%; 27/42, 64%; 23/44, 52%; and 27/42, 64% for levels 1, 2, 3, and 4, respectively). The percentage of men who reported being very satisfied was highest among level 4 participants (10/42, 24% vs 4/39, 10%; 5/42, 12%; and 5/44, 11% for levels 1, 2, and 3, respectively). Dissatisfaction was highest in level 1 (5/39, 13% vs 1/42, 2%; 3/44, 7%; and 2/42, 5% for levels 2, 3, and 4, respectively). We observed small improvements in diet and physical activity at 3 months among men in level 4 versus those in level 1.

**Conclusions:**

A web-based, remotely delivered, tailored behavioral intervention for men with prostate cancer is feasible. Future studies are warranted to increase the effect of the intervention on patient behavior while maintaining sustainability and scalability as well as to design and implement interventions for more diverse populations.

**Trial Registration:**

ClinicalTrials.gov NCT03406013; http://clinicaltrials.gov/ct2/show/NCT03406013

## Introduction

### Background

Prostate cancer affects more than 3.6 million men in the United States, making it the most prevalent cancer in American men [1]. Prostate cancer is characterized by low age-adjusted death rates and a relatively long median survival time of 16 years, although this varies greatly by stage at diagnosis [2]. Over this period, men with prostate cancer may experience significant disease- or treatment-related decline in quality of life [3-6], including incontinence, erectile dysfunction, fatigue, poor metabolic functioning, reduced bone and muscle integrity, hot flushes, sexual dysfunction, and low mood [7].

### Prior Work

A growing number of studies have suggested the benefits of a healthy diet and regular exercise for men with prostate cancer, including lower risk of treatment-associated side effects [8-11], cancer progression [12], and cancer-specific mortality [13-16]. Specific dietary factors that have been associated with improved clinical outcomes in men with prostate cancer include higher intake of cruciferous vegetables, vegetable fat, fish, and cooked tomatoes and lower intake of processed meat, whole milk, and poultry with skin [17-25]. Physical activity recommendations of ≥150 minutes of moderate intensity or ≥75 minutes of high-intensity aerobic exercise per week have also been associated with a lower risk of mortality in men with prostate cancer [26].

Translation of this growing evidence of possible benefits of a healthy lifestyle for prostate cancer survivors into clinical practice and survivorship programs has been slow. Although physical activity and nutrition guidelines exist for cancer survivors [27], lifestyle counseling and exercise programs are not standard care practices for individuals with cancer in the United States. Moreover, most prostate cancer survivors do not follow the recommendations.

Previous studies on lifestyle interventions suggest that center-based interventions are effective but require infrastructure that may only be available in urban academic centers [28]. In contrast, it is challenging to make home-based interventions comprehensive or tailored to the individual needs and interests of the participants [29]. The diverse attitudes and motivations to change behavior among men with prostate cancer further complicate matters. Previous studies suggest that although men with prostate cancer appreciate the importance of exercise, most do not feel that the information provided by their doctor is specific enough to be actionable [30]; many also report low motivation for physical activity [30].

### Objectives

In this context, web-based interventions are emerging as promising, scalable modalities for behavior change [31-36]. Prior literature suggests that tailoring of an intervention to individual characteristics and goals and combining technology with personal guidance (ie, blended intervention) may lead to improved outcomes [37-39]. However, questions regarding the feasibility and acceptability of remotely delivered web-based interventions remain, particularly in older adult populations. Thus, we designed the TrueNTH Community of Wellness study, a 4-arm, multi-site, pilot randomized controlled trial to evaluate the feasibility and acceptability of a 3-month web-based intervention for men with prostate cancer with progressive levels of behavioral support. In this study, we report our primary results on the feasibility and acceptability of the intervention. Second, we describe self-reported levels of physical activity and diet at the time of enrollment and 3 and 6 months after the enrollment and explore changes in lifestyle behaviors over the study period.

## Methods

### Study Design, Population, and Recruitment

We conducted a 4-arm pilot randomized controlled trial of a 3-month intervention among men with prostate cancer (ClinicalTrials.gov NCT03406013) to compare 3 levels of increasing behavioral support (levels 2-4) with general educational information on a website (level 1). The multicenter trial was conducted at Oregon Health Sciences University (OHSU), University of California San Francisco (UCSF), and University of Colorado Denver (UCD).

The trial protocol has been reported [40]. Briefly, men were recruited through hospital cancer registry databases, the Cancer of the Prostate Strategic Urologic Research Endeavor registry of men with prostate cancer, and in clinics. Men were eligible to participate if they self-reported a prostate cancer diagnosis, had a personal device with internet and text messaging capabilities and a personal email address, were aged ≥18 years, and were able to read English. Men currently receiving chemotherapy or radiation therapy or those who had potential contraindications to exercise identified on the basis of the American College of Sports Medicine exercise preparticipation screening criteria could participate with physician clearance.

OHSU was the primary coordinating center for this trial. All participants provided written consent, and all study-related activities were performed in accordance with and under the supervision of the institutional review board of each study site.

### Randomization and Blinding

Consenting men were block randomized (1:1:1:1) by site (UCSF, OHSU, or UCD) to increasing levels of web-based behavioral support (level 1-4). The randomization scheme was computer generated with block size 4 by SZ and stored at UCSF. When a participant had completed consent and enrollment procedures, research staff at OHSU requested the next assignment from UCSF. Participants were told that they would be randomly assigned to different tools and resources but were unaware of which resources they received relative to other participants.

### Intervention Description

Details on the intervention, including its theoretical basis, have been previously reported [40]. Briefly, level 1 (reference group) received general educational information about exercise and diet, a resource directory, and study-specific guidelines delivered through the website (information on the website was not changed throughout the study period for level 1). Level 2 received the information provided to level 1 along with a personalized diet and exercise prescriptions delivered through the website, including videos of recommended exercises and a weekly short survey about their progress toward the diet and exercise recommendations. Level 3 received information and resources provided to level 2 along with a Fitbit Alta (Fitbit Inc) with physical activity reports (Fitbit data integrated into the website), supportive text messages (50 texts over 90 days: average 4 per week, no response required, equally split and alternating between diet and exercise topics), and weekly web-based short surveys for participants to track their progress toward the diet and exercise recommendations. Level 4 received information and resources provided to level 3 as well as 2 optional 30-minute calls: one with an exercise trainer (KD) and one with a registered dietician (GM). Men accessed the study website with a username and password and had to complete an onboarding process to gain access to the intervention website. The study website home page included a dashboard that summarized self-reported diet and physical activity behavior using visuals and contained links to other pages (eg, Report Progress, See Progress, Connect Fitbit, etc), depending on the assigned level. All participants from all levels received an instruction sheet (PDF) orienting them to the website at enrollment and a weekly email reminder to encourage them to use the website.

The intervention recommendations focused on diet and physical activity. The dietary recommendations provided to all levels were to consume one serving each of healthy vegetable fats and cruciferous vegetables per day; 2 servings each of cooked tomatoes and fish per week; and no whole milk, processed meat, or poultry with skin. The individual dietary prescriptions provided to levels 2 to 4 were focused on helping the participants achieve these recommendations, considering what the participants self-reported at baseline. The physical activity recommendations, consistent with national guidelines, were to engage in 150 minutes of aerobic exercise per week, 60 minutes of strength training per week accumulated in ≥2 sessions, and 2 sessions of stretching per week. The individual exercise prescriptions provided to levels 2 to 4 were based on the men’s self-reported current physical activity levels, health goals for exercise, health status, resources for exercise, and time available for exercise. In addition, the exercise prescriptions were tailored to participants’ self-reported current prostate cancer status. Men with bone metastases or active cancer treatment other than androgen deprivation therapy were prescribed low-intensity programs.

### Web-Based Survey Assessments

Participants were asked to complete surveys on the internet at baseline, 3 months (immediately following the intervention), and 6 months using the study website and Research Electronic Data Capture [41]. Surveys included sociodemographics and prostate cancer diagnosis and treatment (baseline only), the Community Health Activities Model Program for Seniors (CHAMPS) [42], and a validated food frequency questionnaire (FFQ) [43]. Each month, the men also completed a web-based survey about adverse events (AEs) and whether any reported AE was related to a pre-existing condition present before enrollment. After 3 months, men were asked to complete a web-based acceptability survey specific to the level of intervention received.

### Primary Outcomes: Feasibility and Acceptability

The primary study outcomes were feasibility and acceptability of the intervention. Feasibility was assessed based on the accrual time and retention. Accrual was defined as the time between enrollment of the first and last participant; our goal was to accrue 200 participants in 12 months. Retention was defined as the proportion of participants who completed at least one of the surveys at each follow-up time point (3 and 6 months). A priori, we specified that we would consider the intervention to be feasible if we retained at least 80% (160/200) of participants at 3 months and 64% (128/200) of participants at 6 months [40].

Acceptability was measured via an investigator-developed web-based survey administered at 3 months and website use. The level-specific survey asked men to assess their overall satisfaction with the program as well as each of the resources received (website, resource directory, exercise prescription, diet prescription, weekly progress report, Fitbit, text messages, diet coach, and exercise coach) as *very satisfied*, *satisfied*, *neutral*, *dissatisfied*, *very dissatisfied*, or *did not use*. To assess overall satisfaction, participants were asked *“*How satisfied were you with your experience with the Community of Wellness intervention and portal?*”* When assessing resources, participants were only asked about resources they received based on their assigned level of intervention. There was also an open text box for additional comments and feedback. The frequency of visits to the study portal was directly assessed using log-ins and web analytic data. A priori, we expected all men to log onto the website at least once and that the frequency of log-ins would increase across levels from level 1 to 4 [40]. There was a delay in activating the analytics function between August and December 2017; therefore, there were no data for the first 7 enrollees and partial data for those enrolled between October and December 2017. Thus, summaries of web analytics data reflect an underestimate of use.

### Secondary Outcomes: Change and Maintenance of Diet and Physical Activity

The intervention was designed to support the adoption or maintenance of diet and physical activity habits previously reported to be associated with reducing the risk of prostate cancer recurrence or mortality. Thus, we estimated the effect of the intervention levels on changes in self-reported diet and physical activity as secondary outcomes.

### Diet Assessment

We used a validated FFQ to assess the usual diet at the time of enrollment and 3 and 6 months after the enrollment [43]. The FFQ asked men to report their usual intake of approximately 140 foods and beverages over the past 3 months, with up to nine frequency options ranging from never or <1 per month to ≥6 per day. *Cruciferous vegetables* included a half cup of broccoli, cauliflower, cabbage, brussels sprouts, kale, mustard greens, or chard*. Vegetable fat* included avocado (half cup), oil dressing (1-2 tbs), peanut butter (1 tbs), peanuts (1 oz), walnuts (1 oz), and other nuts (1 oz). *Cooked tomatoes* included intake of tomato sauce (half cup), salsa (quarter cup), and pizza (2 slices). *Fish* included tuna fish (3-4 oz), fish sticks (1 serving), dark meat fish (eg, mackerel, salmon, sardines, bluefish, swordfish; 3-5 oz), and other fish (3-5 oz). *Processed meat* included intake of beef or chicken hotdogs (1 hotdog), bacon (2 slices), processed meat sandwiches (eg, salami, bologna), and other processed meat (eg, sausage, kielbasa; 2 oz or 2 small links). Consumption of *whole milk* was assessed by asking men how frequently they consumed 1 glass (8 oz) of whole milk. Consumption of *poultry with skin* was assessed by asking men how frequently they consumed 3 to 4 oz of chicken or turkey with skin. FFQs with >70 items blank were considered incomplete (31 participants at 3 months and 36 participants at 6 months); these data were not included in secondary analyses examining dietary change.

### Physical Activity Assessment

We used the CHAMPS survey to quantify 3 types of physical activities: aerobic, strength training, and stretching. *Aerobic activity* included minutes per week (min/wk) of moderate-to-vigorous aerobic activities, including tennis, skating (ice, roller, or inline), jogging or running, walking or hiking uphill, walking fast for exercise, riding bikes, aerobic machines (eg, rowing, step), swimming, water exercises, aerobics or dancing, and sports (eg, basketball, soccer, racquetball). We calculated the number of sessions and total time per week spent doing *strengthening activities* and the number of weekly s*tretching* sessions, as reported in the CHAMPS survey [42]. If a participant responded to at least one item on the CHAMPS survey, items that were skipped were assumed to be zero. If a participant did not respond to any question, his physical activity was missing for that time point (3 men at baseline, 49 men at 3 months, and 57 men at 6 months).

### Total Lifestyle Behavior Score

To combine diet and physical activity into one measure, we developed a total lifestyle behavior score [40]. Multimedia Appendix 1 lists the items and points assigned to calculate the score. Men were given points for each component: 0 (not meeting recommendation), 1 (almost meeting recommendation), or 2 (meeting recommendation). Scores were summed across components to develop a diet score ranging from 0 to 14, with 14 assigned to men fully meeting dietary recommendations; a physical activity score ranging from 0 to 6, with 6 assigned to men fully meeting the physical activity recommendations; and an overall lifestyle score ranging from 0 to 20, with a score of 20 assigned to men fully meeting all lifestyle recommendations. One recommendation (ie, only taking supplements that have been reviewed with a health professional) was unable to be included in the score [40], as the question was inadvertently omitted from the 3-month survey following a switch in the technology platform after the initial 20 participants.

### Statistical Analysis

Descriptive statistics, including proportions for categorical variables and median (IQR) for continuous variables, were used to summarize the characteristics of the participants, overall and by level of intervention received. We also used descriptive statistics to describe responses to the acceptability survey. Two participants originally randomized to level 1 and level 2 were incorrectly provided access to the level 3 intervention. In our primary acceptability and feasibility analyses, we analyzed these individuals based on intervention received (level 3), as acceptability surveys and web analytics were specific to level received. To compute participation proportions and secondary analyses of behavior change and AEs, the men were analyzed using the original randomization level (level 1 and level 2).

To explore diet and physical activity behaviors, we calculated the median (IQR) dietary score, physical activity score, and total lifestyle behavior score at each time point. Summary statistics for each component of diet and physical activity scores per arm of the study at each time point were also reported. We used *t* tests to calculate the mean change and 95% CI between baseline and 3 months and baseline and 6 months for each of the scores, within and between arms. As these were secondary analyses, we used a complete case approach and described levels of diet and physical activity at each time point among participants with available data.

In a posthoc sensitivity analysis, we also examined aerobic activity at each time point by level and aerobic physical activity at enrollment. We hypothesized that men with low levels of physical activity at enrollment would increase their activity at 3 months, whereas there would be no change among men who were already performing the recommended 150 minutes per week of physical activity.

Analyses were performed using Stata version 16.0 (StataCorp LP). As specified in our protocol, we tested for differences in change in the 3 behavior scores across arms using the standard α level of .05 to assess statistical significance. For the rest of our analyses, we adhered to the Consolidated Standards of Reporting Trials recommendation for pilot and feasibility studies and report descriptive statistics only [44].

## Results

### Recruitment and Retention

**Figure 1:**
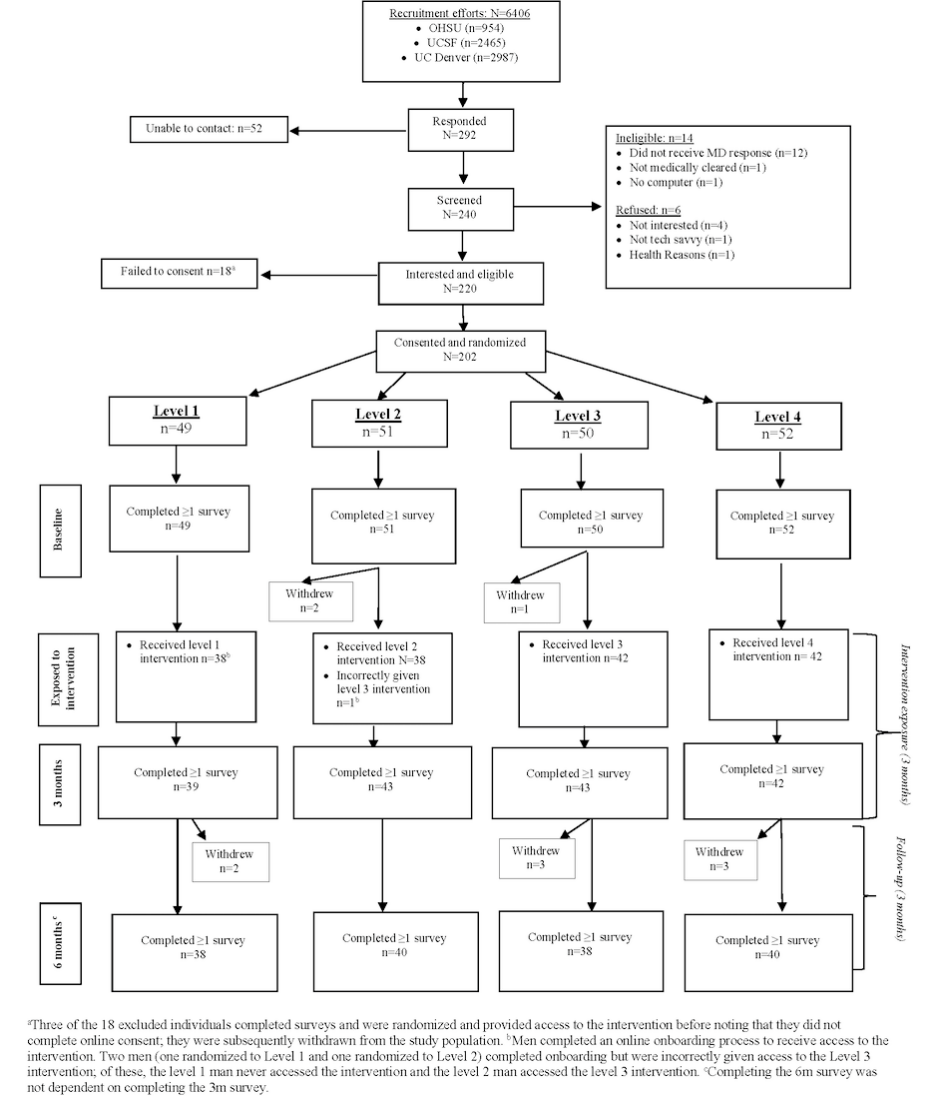
Consolidated Standards of Reporting Trials (CONSORT) diagram showing the flow of participants from screening through end of study. MD: medical doctor; OHSU: Oregon Health and Sciences University; UC Denver: University of Colorado Denver; UCSF: University of California San Francisco.

A total of 6406 men received a letter, between July 2017 and September 2018, detailing the study and providing contact information for those who wanted to learn more (Figure 1). Of these, 292 men expressed interest, 240 were screened for eligibility, and 220 were interested and deemed eligible. The main reason for ineligibility was not receiving medical clearance (13 men). Furthermore, 1 man did not own a computer and 6 declined to participate after screening. Of the 220 interested and eligible men, 202 were randomized and provided access to the web-based consenting process and surveys. These 202 men comprise the initial population for analysis.

The 202 men with prostate cancer were randomized (1:1:1:1) to increasing levels of web-based behavioral support; 49 were assigned to level 1, 51 were assigned to level 2, 50 were assigned to level 3, and 52 were assigned to level 4 (Figure 1). Of the 202 randomized men, 161 completed the onboarding process and were exposed to the intervention. Of these men, 160 were exposed to their randomly assigned intervention (38 in level 1, 38 in level 2, 42 in level 3, and 42 in level 4). As noted above, 2 men were incorrectly given access to level 3, when originally assigned to levels 1 and 2; only one of these men (originally assigned to level 2) initiated the intervention by accessing the study website. Throughout the 6-month follow-up, 11 men withdrew after randomization; the primary reason for withdrawal was the time commitment. Withdrawal was similar across groups; 2 men withdrew in level 1, 2 men withdrew in level 2, 4 men withdrew in level 3, and 3 men withdrew in level 4.

Overall, 82.7% (167/202) and 77.2% (156/202) of men completed surveys after 3 and 6 months, respectively. By level, the 3- and 6-month follow-up proportions were 80% (39/49) and 78% (38/ 49) for level 1, 84% (43/ 51) and 78% (40/ 51) for level 2, 86% (43/ 50) and 76% (38/50) for level 3, and 81% (42/52) and 77% (40/52) for level 4 (Figure 1).

### Characteristics of the Study Population

Of the 202 randomized participants, the men were predominantly White (187/202, 92.6%), well-educated (167/202, 82.7%, reported a 4-year college degree or more), and married or had a partner (185/202, 91.6%; Table 1). The median (IQR) age at enrollment was 70 years (65-75 years) and the median BMI was 27 kg/m^2^ (25-29 kg/m^2^). Participants self-reported a wide spectrum of prostate cancer stages and grades. The median (IQR) prostate-specific antigen at diagnosis was 6 ng/ml (5-10 ng/ml), 13.9% (28/202) reported T3 disease, and 5.9% (12/202) reported T4 disease; 39.6% (80/202) had intermediate grade (Gleason sum 7) and 22.8% (46/202) had high grade (Gleason sum 8-10) cancer. Most participants reported that they had been treated for localized disease at the time of enrollment or were on active surveillance. The characteristics of the men assigned to each of the 4 levels were similar.

**Table 1:** Baseline self-reported sociodemographic and clinical characteristics of 202 men with prostate cancer participating in a technology-supported physical activity and dietary intervention, overall and by level randomized.

Characteristics^a^		Level 1	Level 2	Level 3	Level 4	Total
Number of men, n (%)		49 (24.3)	51 (25.2)	50 (24.8)	52 (25.7)	202 (100.0)
Age (years), median (IQR)		70 (64-76)	70 (64-75)	70 (64-75)	70 (65-74)	70 (65-75)
BMI (kg/m^2^), median (IQR)		25 (23-28)	28 (26-30)	26 (24-29)	27 (25-29)	27 (25-29)
**Race, n (%)**						
	White	44 (90)	45 (88)	48 (96)	50 (96)	187 (92.6)
	Black	2 (4)	3 (6)	0 (0)	0 (0)	5 (2.5)
	Other	0 (0)	1 (2)	0 (0)	0 (0)	1 (0.5)
	Asian	1 (2)	0 (0)	0 (0)	1 (2)	2 (1.0)
	More than one race	2 (4)	2 (4)	0 (0)	1 (2)	5 (2.5)
	Decline to answer	0 (0)	0 (0)	2 (4)	0 (0)	2 (1.0)
**Education, n (%)**						
	Grade school	0 (0)	1 (2)	0 (0)	0 (0)	1 (0.5)
	High school	4 (8)	6 (12)	1 (2)	3 (6)	14 (6.9)
	2-year college	9 (18)	4 (8)	2 (4)	5 (10)	20 (9.9)
	4-year college	10 (20)	14 (27)	20 (40)	14 (27)	58 (28.7)
	Graduate or professional degree	26 (53)	26 (51)	27 (54)	30 (58)	109 (54.0)
**Employment, n (%)**						
	Decline to answer	0 (0)	0 (0)	0 (0)	1 (2)	1 (0.5)
	Full time	12 (24)	11 (22)	17 (34)	10 (19)	50 (24.8)
	Part time	6 (12)	2 (4)	5 (10)	8 (15)	21 (10.4)
	Retired	31 (63)	37 (73)	27 (54)	31 (60)	126 (62.4)
	Disabled	0 (0)	0 (0)	0 (0)	1 (2)	1 (0.5)
	Unemployed	0 (0)	1 (2)	1 (2)	1 (2)	3 (1.5)
**Marital status, n (%)**						
	Married or partnered	46 (94)	46 (90)	46 (92)	47 (90)	185 (91.6)
	Divorced or separated	1 (2)	1 (2)	1 (2)	3 (6)	6 (3.0)
	Widowed	1 (2)	2 (4)	2 (4)	0 (0)	5 (2.5)
	Never married	0 (0)	2 (4)	1 (2)	2 (4)	5 (2.5)
	Decline to answer	1 (2)	0 (0)	0 (0)	0 (0)	1 (0.5)
PSA^b^ at Dx^c^ (ng/ml), median (IQR)		5 (4-10)	7 (5-11)	7 (5-10)	6 (5-12)	6 (5-10)
**T-stage^d^, n (%)**						
	T1	15 (31)	15 (30)	13 (26)	19 (37)	62 (30.7)
	T2	25 (51)	19 (38)	29 (58)	14 (27)	87 (43.1)
	T3	5 (10)	12 (24)	5 (10)	6 (12)	28 (13.9)
	T4	1 (2)	2 (4)	3 (6)	6 (12)	12 (5.9)
	Unknown	3 (6)	2 (4)	0 (0)	7 (13)	12 (5.9)
**Gleason sum at Dx, n (%)**						
	5, low grade	0 (0)	0 (0)	1 (2)	3 (6)	4 (2.0)
	6, low grade	6 (12)	7 (14)	13 (26)	8 (15)	34 (16.8)
	3+4, intermediate grade	16 (33)	5 (10)	10 (20)	10 (19)	41 (20.3)
	4+3, intermediate grade	10 (20)	12 (24)	11 (22)	6 (12)	39 (19.3)
	8-10, high grade	8 (16)	14 (28)	12 (24)	12 (23)	46 (22.8)
	Unknown or Do not know	9 (18)	12 (24)	3 (6)	13 (25)	37 (18.3)
**Disease status at enrollment^e^, n (%)**						
	On active surveillance, PSA low or not-rising	3 (6)	7 (14)	3 (6)	6 (12)	19 (9.4)
	On active surveillance, PSA elevated or rising	1 (2)	2 (4)	5 (10)	4 (8)	12 (5.9)
	Completed treatment for localized disease, PSA low or undetectable	31 (63)	23 (45)	26 (52)	27 (52)	107 (53.0)
	Completed treatment for localized disease, PSA elevated	3 (6)	1 (2)	4 (8)	2 (4)	10 (5.0)
	Cancer spread locally	1 (2)	2 (4)	1 (2)	3 (6)	7 (3.5)
	Metastatic disease	2 (4)	5 (10)	4 (8)	3 (6)	14 (6.9)
	Other or Do not know	8 (16)	11 (22)	7 (14)	7 (13)	33 (16.3)
Time since Dx (years)^f^, median (IQR)		9 (4-14)	4 (2-7)	3 (2-10)	4 (2-9)	4 (2-10)
Time since first treatment (years), median (IQR)		2 (1-9)	4 (2-10)	3 (1-11)	3 (1-6)	3 (1-9)
**Enrollment site, n (%)**						
	Oregon (OHSU^g^)	21 (43)	23 (45)	15 (30)	18 (35)	77 (38.1)
	California (UCSF^h^)	16 (33)	14 (27)	19 (38)	19 (37)	68 (33.7)
	Colorado (UCD^i^)	12 (24)	14 (27)	16 (32)	15 (29)	57 (28.2)

^a^Percentages may not sum to 100% due to rounding.

^b^PSA: prostate-specific antigen.

^c^Dx: diagnosis.

^d^T-Stage: Tumor (T) component of the TNM staging system developed by the American Joint Committee on Cancer.

^e^Self-reported disease status.

^f^Diagnosis date was known for 127 men (31, 30, 32, 34 in levels 1-4), and the first treatment date was known for 135 men (26, 34, 39, 36 in levels 1-4).

^g^OHSU: Oregon Health Sciences University.

^h^UCSF: University of California San Francisco.

^i^UCD: University of Colorado Denver.

### Acceptability of Intervention

As hypothesized, engagement and satisfaction with the intervention increased across levels (Table 2). On the basis of web analytics data on 154 of 161 men exposed to the intervention, the median (IQR) number of days that participants went to the study website over the 3-month intervention was 2 (IQR 1-3) in level 1, 9 (IQR 5-13) in level 2, 11 (IQR 8-16) in level 3, and 16 (IQR 10-19) in level 4. The most commonly visited pages for men in levels 2 to 4 were the main home page/dashboard and the follow-up survey page where participants could self-report progress toward their diet and exercise goals. Although all other pages were visited at least once, the median number of days that participants visited the remaining pages was low (1-5 visits each).

**Table 2:** Acceptability of a 3-month technology-supported behavioral intervention for men with prostate cancer by level of intervention received.

Characteristics^a^		Level 1	Level 2	Level 3	Level 4
Number of men^b^		39	42	44	42
Frequency of portal visits (days), median (IQR)^b^		2 (1-3)	9 (5-13)	11 (8-16)	16 (10-19)
**Overall satisfaction with Community of Wellness intervention and portal, n (%)**					
	Very satisfied	4 (10)	5 (12)	5 (11)	10 (24)
	Satisfied	16 (41)	22 (52)	18 (41)	17 (40)
	Neutral	10 (26)	11 (26)	15 (34)	11 (26)
	Dissatisfied	4 (10)	1 (2)	2 (5)	1 (2)
	Very dissatisfied	1 (3)	0 (0)	1 (2)	1 (2)
	Did not report	4 (10)	3 (7)	3 (7)	2 (5)
**Resource directory, n (%)**					
	Excellent	1 (3)	2 (5)	0 (0)	4 (10)
	Very good	5 (13)	9 (21)	4 (9)	6 (14)
	Good	12 (31)	9 (21)	14 (32)	8 (19)
	Average	3 (8)	3 (7)	5 (11)	7 (17)
	Poor	0 (0)	2 (5)	0 (0)	3 (7)
	Did not use	13 (33)	12 (29)	18 (41)	10 (24)
	Did not report	5 (13)	5 (12)	3 (7)	4 (10)
**Exercise prescription, n (%)**					
	Excellent	—^c^	1 (2)	3 (7)	10 (24)
	Very good	—	11 (26)	6 (14)	10 (24)
	Good	—	13 (31)	19 (43)	10 (24)
	Average	—	5 (12)	6 (14)	5 (12)
	Poor	—	0 (0)	0 (0)	1 (2)
	Did not use	—	4 (10)	6 (14)	4 (10)
	Did not report	—	8 (19)	4 (9)	2 (5)
**Diet prescription, n (%)**					
	Excellent	—	2 (5)	6 (14)	12 (29)
	Very good	—	11 (26)	11 (25)	12 (29)
	Good	—	12 (29)	12 (27)	4 (10)
	Average	—	5 (12)	3 (7)	8 (19)
	Poor	—	0 (0)	2 (5)	0 (0)
	Did not use	—	4 (10)	6 (14)	3 (7)
	Did not report	—	8 (19)	4 (9)	3 (7)
**Weekly progress report, n (%)**					
	Excellent	—	2 (5)	3 (7)	11 (26)
	Very good	—	15 (36)	12 (27)	13 (31)
	Good	—	6 (14)	14 (32)	6 (14)
	Average	—	4 (10)	7 (16)	6 (14)
	Poor	—	1 (2)	1 (2)	1 (2)
	Did not use	—	6 (14)	3 (7)	2 (5)
	Did not report	—	8 (19)	4 (9)	3 (7)
**Fitbit, n (%)**					
	Excellent	—	—	9 (20)	6 (14)
	Very good	—	—	5 (11)	8 (19)
	Good	—	—	7 (16)	7 (17)
	Average	—	—	3 (7)	1 (2)
	Poor	—	—	8 (18)	4 (10)
	Did not use	—	—	7 (16)	12 (29)
	Did not report	—	—	5 (11)	4 (10)
**Text messages, n (%)**					
	Excellent	—	—	0 (0)	4 (10)
	Very good	—	—	4 (9)	8 (19)
	Good	—	—	14 (32)	8 (19)
	Average	—	—	10 (23)	2 (5)
	Poor	—	—	1 (2)	5 (12)
	Did not use	—	—	10 (23)	11 (26)
	Did not report	—	—	5 (11)	4 (10)
**Diet coach, n (%)**					
	Excellent	—	—	—	9 (21)
	Very good	—	—	—	9 (21)
	Good	—	—	—	6 (14)
	Average	—	—	—	6 (14)
	Poor	—	—	—	1 (2)
	Did not use	—	—	—	7 (17)
	Did not report	—	—	—	4 (10)
**Exercise coach, n (%)**					
	Excellent	—	—	—	8 (19)
	Very good	—	—	—	9 (21)
	Good	—	—	—	6 (14)
	Average	—	—	—	7 (17)
	Poor	—	—	—	2 (5)
	Did not use	—	—	—	6 (14)
	Did not report	—	—	—	4 (10)

^a^Percentages may not sum to 100% due to rounding.

^b^Web analytics data were available for 154 (33, 38, 41, and 42 for levels 1, 2, 3, and 4, respectively) of 161 men exposed to the intervention. The acceptability survey was sent after the 3-month intervention period; because of technical errors, 44 men received the acceptability survey late.

^c^Empty cells denote intervention features that were not offered to a given level; participants were not asked to provide feedback on features they did not receive.

A higher proportion of men (10/42, 24%) in level 4 reported that they were very satisfied with the intervention than men in levels 1, 2, and 3 (4/39, 10%; 5/42, 12%; and 5/44, 11%, respectively)*.* A total of 6 men in levels 1 to 3 (1 in level 1, 4 in level 2, and 1 in level 3) who completed surveys at 3 months had not accessed the study website intervention (Figure 1), which may in part reflect lower satisfaction scores for these levels compared with level 4. In addition, a higher proportion of men in level 1 reported that they were dissatisfied or very dissatisfied with the intervention (5/39, 13%) compared with levels 2, 3, and 4 (1/42, 2%; 3/44, 7%; and 2/42, 5%, respectively). Men in level 4 (n=42) were also more likely to rate the intervention features as excellent or very good: exercise prescription (20/42, 48%), diet prescription (24/42, 57%), and weekly progress report (24/42, 57%) compared with the 42 men in level 2 (12/42, 29%; 13/42, 31%; 17/42, 40%, respectively), and 44 men in level 3 (9/44, 20%; 17/44, 39%; 15/44, 34%). In contrast to our expectations, a large number of men in levels 3 and 4 reported that they did not like (8/44, 18% and 4/42, 10%, respectively) or did not use (7/44, 16% and 12/42, 29%, respectively) the Fitbit. Approximately one-fourth of the men in levels 3 and 4 reported that they did not use the text messages (10/44, 23% in level 3 and 11/42, 26% in level 4). Of the 42 men in level 4, 26 (62%) completed an exercise coaching call (8 were unable to be contacted and 8 declined) and 35 (83%) completed a diet coaching call (2 were unable to be contacted and 5 declined). Of the men who received the calls, 88% (23/26) rated the exercise call as good to excellent and 69% (24/35) rated the diet call as good to excellent.

Participants’ open-ended feedback on the intervention is provided in Multimedia Appendix 2. Participants noted challenges with the onboarding process, insufficient personalization of the study materials, and limited information tailored to their level and readiness for change. Although the intervention provided personalized diet and exercise prescriptions based on baseline surveys (levels 2-4), feedback from the open-ended comments indicated that several participants desired more interactive feedback, direction, and reminders. As noted above, many men indicated that they did not like or use the Fitbit; some participants already owned other devices and would have preferred to have the option to integrate those into the study website.

### Lifestyle Behavior

**Figure 2:**
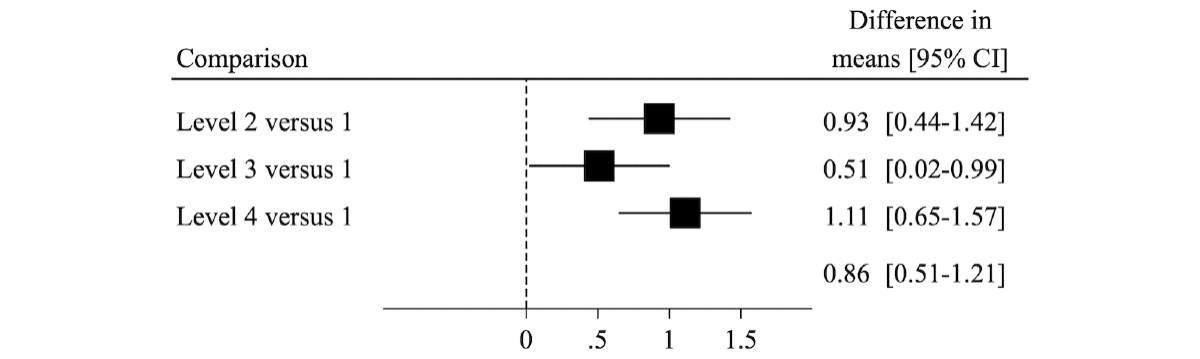
Difference in mean change in an overall lifestyle score (range 0-20) from baseline to 3 months, comparing intervention levels 2 to 4 with level 1. Higher scores indicate more healthy lifestyle behaviors. These secondary analyses included the 146 men with complete data on lifestyle behaviors at 0 and 3 months.

**Figure 3:**
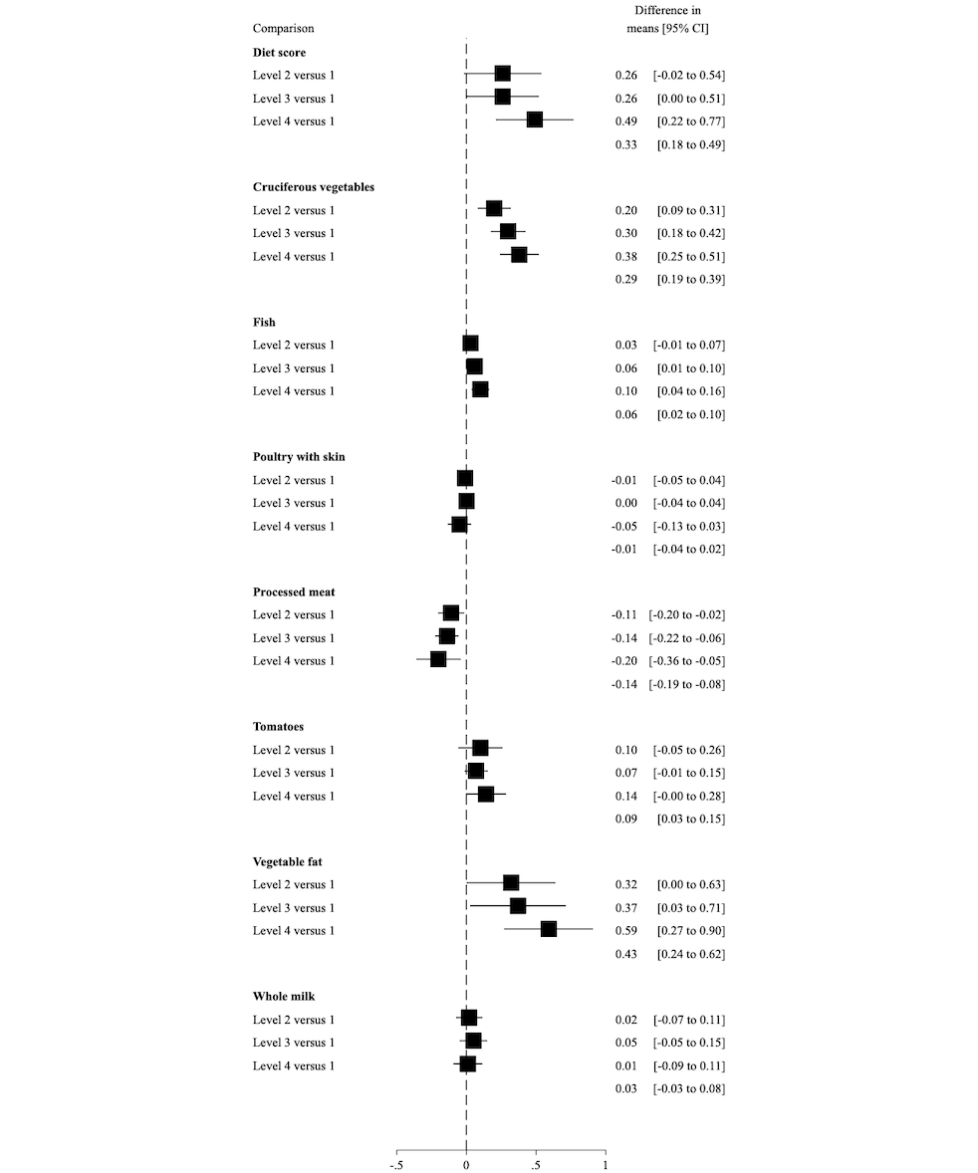
Differences in mean change in an overall diet score (range 0-14) and servings per day of diet items from baseline to 3 months, comparing intervention levels 2 to 4 with level 1. A higher score for the overall diet score indicates healthier diet behaviors. The intervention aimed to increase intake of cruciferous vegetables, fish, tomatoes, and vegetable fat and decrease intake of poultry with skin, processed meat, and whole milk. These secondary analyses included the 146 men with complete data on diet behaviors at 0 and 3 months.

**Figure 4:**
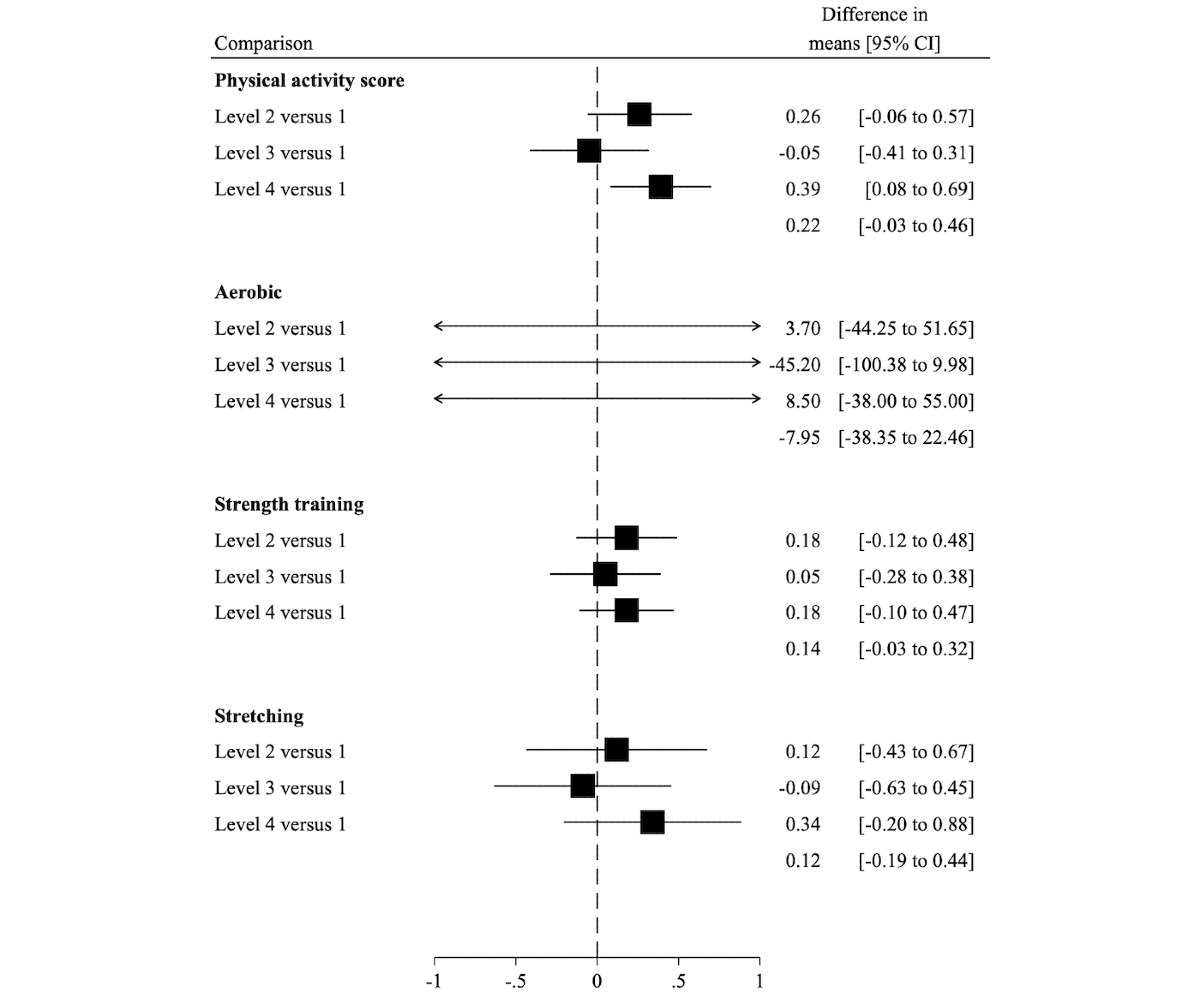
Difference in mean change in an overall physical activity score (range 0-6) and types of physical activity (minutes per week of aerobic; sessions per week of strength training and stretching) from baseline to 3 months, comparing intervention levels 2 to 4 with level 1. Higher scores indicate more physical activity. These secondary analyses were performed among the 152 men with complete data on physical activity at 0 and 3 months.

Figure 2 shows the between-group difference in mean change (95% CI) from enrollment to 3 months comparing with the 4 levels for the overall lifestyle score. Changes between levels 2, 3, and 4 versus level 1 for the diet and physical activity scores and their subcomponents are shown in Figure 3 and Figure 4. The between-group differences in mean change, comparing each level to level 1, were as follows: 0.93 (95% CI 0.44-1.41) points for level 2; 0.51 (95% CI 0.02-0.99) points for level 3; and 1.11 (95% CI 0.65-1.57) points for level 4. The median (IQR) values for the lifestyle score and its components, by study arm and time point, are shown in Multimedia Appendix 3 and the between-group differences in mean change from enrollment to 6 months are shown in Multimedia Appendices 4-6. The small improvement in score for level 4 was attenuated, but still present, at 6 months (between-group difference in mean change level 4 vs 1: 0.72; 95% CI 0.26-1.18 points).

Level 4 had a greater improvement in diet scores at 3 months compared with level 1 (Figure 3). The between-group difference in mean change from enrollment to month 3 in level 4 as compared with level 1 was 0.49 (IQR 0.22-0.77) points. This change seemed to be driven by an increased intake of cruciferous vegetables and healthy vegetable fat and decreased intake of processed meat. As with the overall lifestyle score, the improvement was present, but attenuated, at 6 months (between-group difference in mean change in level 4 vs level 1: 0.32 [IQR 0.09-0.54]; Multimedia Appendix 5).

Similarly, for physical activity, only level 4 appeared to have a greater increase in physical activity at 3 months compared with level 1 (between-group difference in mean: 0.39; 95% CI 0.08-0.69 points; Figure 4). The change appeared to be due to small increases in aerobic exercise, strength training, and stretching. However, the difference in mean change for the overall score was not maintained at 6 months (Multimedia Appendix 6).

In a *posthoc* sensitivity analysis, we stratified men according to their self-reported aerobic activity level at the time of enrollment. As shown in Table 3, in levels 3 and 4, men who reported <90 min/wk of aerobic physical activity at enrollment appeared to report more aerobic physical activity at 3 months (median change from baseline to 3 months in level 3: 60 min/wk; IQR 30-75; level 4: 75 min/wk; IQR 30-150). Although these changes were not fully maintained, men in levels 3 and 4 who started with <90 min/wk of aerobic activity reported a median increase of 30 min/wk at 6 months compared with enrollment (level 3 IQR 0-135; level 4 IQR 0-330).

**Table 3:** Moderate-to-vigorous intensity aerobic physical activity minutes per week at baseline, 3 months, and 6 months among men with prostate cancer participating in a technology-supported behavioral intervention, overall and by level randomized and baseline activity.

Time point^a^		Level 1		Level 2		Level 3		Level 4	
		n	Median (IQR)	n	Median (IQR)	n	Median (IQR)	n	Median (IQR)
≥**150 min/week at enrollment**									
	Baseline	30	345 (225 to 480)	22	405 (225 to 540)	33	345 (240 to 510)	28	398 (255 to 480)
	3 months	22	263 (210 to 495)	16	323 (173 to 495)	28	285 (105 to 450)	21	435 (330 to 540)
	6 months	22	398 (105 to 540)	15	345 (135 to 570)	26	383 (225 to 465)	20	338 (315 to 405)
	Change, baseline to 3 months	22	−75 (−180 to 90)	16	15 (−263 to 128)	28	−53 (−360 to 75)	21	−75 (−195 to 180)
	Change, baseline to 6 months	22	−38 (−150 to 120)	15	0 (−150 to 315)	26	−8 (−120 to 75)	20	−165 (−285 to 120)
**90-<150 min/week at enrollment**									
	Baseline	6	105 (105 to 105)	9	105 (105 to 105)	3	120 (105 to 135)	10	105 (105 to 105)
	3 months	4	398 (203 to 570)	8	120 (68 to 188)	3	225 (105 to 330)	8	150 (83 to 233)
	6 months	4	180 (83 to 233)	8	180 (45 to 315)	3	105 (0 to 390)	8	173 (98 to 225)
	Change, baseline to 3 months	4	293 (98 to 465)	8	15 (−38 to 90)	3	105 (−30 to 225)	8	45 (−23 to 120)
	Change, baseline to 6 months	4	75 (−23 to 128)	8	75 (−60 to 210)	3	0 (−135 to 270)	8	68 (0 to 120)
**<90 min/week at enrollment**									
	Baseline	13	30 (0 to 30)	18	15 (0 to 30)	14	0 (0 to 30)	13	30 (0 to 30)
	3 months	10	45 (0 to 135)	14	30 (0 to 225)	9	105 (30 to 105)	9	105 (30 to 210)
	6 months	10	98 (0 to 105)	13	60 (0 to 105)	7	30 (0 to 135)	9	60 (0 to 360)
	Change, baseline to 3 months	10	0 (0 to 105)	14	30 (0 to 195)	9	60 (30 to 75)	9	75 (30 to 150)
	Change, baseline to 6 months	10	53 (0 to 75)	13	0 (0 to 105)	7	30 (0 to 135)	9	30 (0 to 330)

^a^Two men in level 2 and 1 man in level 4 had unknown baseline physical activity.

### Nonserious Adverse Events

No serious AEs occurred during the study. However, total nonserious AEs (study related and unrelated) were common in the older population of men with prostate cancer (Table 4). Most of the AEs (246/356, 69.1%) were associated with a pre-existing condition; only 15.4% (55/356) of the AEs were self-reported by the participants to be related to the study and included exacerbations of pre-existing problems. Muscle pain/injury, fatigue, and joint or bone pain were the most frequently reported, accounting for 82% (45/55) of study-related AEs. All study-related AEs were mild to moderate in severity. Although the number of study-related AEs was low, they did appear to increase across levels, particularly going from level 1 (8 study-related AEs) to level 2 (14 study-related AEs). The difference in study-related AEs across levels was driven by higher reports of muscle pain/injury and fatigue in higher levels. For example, there were 7 reports of muscle pain/injury that were deemed study related by participants in levels 3 and 4, 6 in level 2, and 3 in level 1. Study-related fatigue was highest in level 4, reported 6 times, compared with 2 reports of fatigue in level 1 and 1 report of fatigue in levels 2 and 3 each.

**Table 4:** Nonserious adverse events self-reported at 3 months among men with prostate cancer participating in a technology-supported behavioral intervention by randomized level.

Nonserious AEs^a^			Level 1		Level 2		Level 3		Level 4		Total	
Total AE, n			80		133		76		67		356	
AE related to pre-existing conditions, n			53		91		54		48		246	
Study-related AE, n			8		14		15		18		55	
**Specific AEs, n**												
*.*	**Joint or bone pain**											
		Any		21		40		24		20		105
		Pre-existing		16		31		20		15		82
		Study related		1		5		5		1		12
	**Muscle pain or injury**											
		Any		20		29		24		15		88
		Pre-existing		9		17		14		9		49
		Study related		3		6		7		7		23
	**Gastrointestinal issues**											
		Any		14		15		8		11		48
		Pre-existing		11		12		5		9		37
		Study related		2		1		2		3		8
	**Fatigue**											
		Any		14		24		10		16		64
		Pre-existing		9		17		10		11		47
		Study related		2		1		1		6		10
	**Dizziness or vertigo**											
		Any		9		15		8		3		35
		Pre-existing		6		9		3		2		20
		Study related		0		1		0		0		1
	**Shortness of breath**											
		Any		1		8		2		2		13
		Pre-existing		1		4		2		2		9
		Study related		0		0		0		1		1
	**Cardiovascular event**											
		Any		1		2		0		0		3
		Pre-existing		1		1		0		0		2
		Study related		0		0		0		0		0

^a^AE: adverse event.

## Discussion

### Principal Findings

The TrueNTH Community of Wellness study primarily sought to evaluate whether persons living with a diagnosis of prostate cancer would engage with a web-based intervention focused on diet and physical activity and secondarily explored whether such an intervention would help people adopt healthier habits. This national, multi-site, pilot randomized controlled trial demonstrated the feasibility of a technology-enhanced, remotely delivered behavioral intervention and provides insights into the acceptability of different intervention components. Overall, we met our a priori goals to enroll 200 participants in 1 year (202 men were randomized in 13 months) and retain 80% of participants at 3 months and 64% at 6 months (retention was 167/202, 82.7% and 156/202, 77.2% at 3 and 6 months, respectively).

Key takeaways from this study include the importance of an easy *onboarding* process and the value of at least some contact from a person. A high proportion of men reported difficulty registering on the portal website, and a quarter of the men failed to complete the process and did not end up receiving their assigned intervention. This underscores the need for additional orientation, email reminders, and/or follow-up calls to ensure that participants have sufficient technical support to access web-based resources. Future studies need to address how to collect sufficient personal information to deliver a tailored intervention without overburdening participants and preventing them from continuing in the program. In addition, individuals who received two 30-minute coach calls (level 4) were more satisfied with the intervention compared with other groups and appeared to be more successful in making small lifestyle changes. These results suggest that some level of coaching or human interaction is important for participant satisfaction with remotely delivered lifestyle programs. This is in agreement with previous findings [45-47]. Additional research is needed to assess whether other technology-based interactions (eg, tailored text messages or chatbots) or web-based peer-to-peer interactions can facilitate similar satisfaction and improve behavior change outcomes as coaching calls [47-50]. Overall, future studies are challenged to identify the minimum dose of health coaching needed for participant satisfaction and meaningful behavior change while maintaining scalability. These studies should consider innovative study designs that efficiently support testing multiple intervention components (eg, multiphase optimization strategy framework) [51,52].

In secondary analyses, it appeared that the intervention had small effects on lifestyle behaviors. For example, we observed a median increase of 0.5 servings per day (IQR 0.2-0.9) in cruciferous vegetable consumption in level 4 at 3 months. The changes we observed are of similar magnitude to those reported by other studies with more intensive health coaching. The Reach Out to Enhance Wellness trial conducted among long-term breast, prostate, and colorectal cancer survivors included 15 health coaching sessions over 12 months and observed an increase in *fruit and vegetable* intake of 1.1 servings per day (95% CI 0.76-1.47) when comparing the intervention group with the control group at 12 months [53]. Our results comparing the different intervention levels add to the literature and suggest that at least some *higher touch* coaching may be needed to successfully modify dietary intake.

Most men in the study did not increase their physical activity from the time of enrollment. Our study population, however, reported high levels of physical activity at enrollment and thus did not have much room for improvement. Indeed, when we stratified men based on whether they met the recommended amounts of physical activity at enrollment, we observed an increase in aerobic physical activity at 3 months among men not meeting the physical activity guidelines at baseline in levels 3 and 4. For example, the median (IQR) min/wk of moderate-to-vigorous physical activity at enrollment and 3 months among inactive men in level 4 was 30 (IQR 0-30) and 105 (IQR 30-210). This change was not maintained at 6 months. Among those who had already met the recommended 150 minutes of exercise per week at the time of enrollment, there was no increase in exercise with increasing intervention levels, and among those assigned to levels 3 and 4, there appeared to be a decrease in total minutes of exercise. It is possible that very active men altered their behavior once they realized that they exceeded the guidelines. Future studies using adaptive trial designs, such as sequential multiple assignment randomized trials, to target intervention resources to participants who need them most would be of interest.

This trial was designed to evaluate the feasibility of direct-to-patient enrollment and acceptability of a remotely delivered, web-based behavioral intervention in a study population with a wide distribution of geography and clinical disease features. Our team previously reported the results of the Prostate 8-I (P8-I) pilot study conducted at UCSF. P8-I reported a larger improvement in diet than that observed in the present trial. One difference in study design that may have played a role in the different results could be the in-clinic recruitment and on-site study visits in P8-I. Given the difference in acceptability reported by men in level 4 compared with levels 1 to 3 in this study, it is possible that having a personal connection to the study helped motivate participants in P8-I to make larger dietary changes. In addition, P8-I excluded participants who had already met 4 of 8 prespecified lifestyle recommendations, whereas this pilot did not.

### Limitations

There are several limitations of this study to consider and improve upon in future trials. First, individuals who volunteered for this study were predominantly White and highly educated; thus, our results may not be generalizable to all men with prostate cancer. Further work is warranted to assess whether a remotely delivered, web-based intervention is acceptable or beneficial for more diverse or underserved populations. Most participants also had localized disease; the feasibility and acceptability of lifestyle interventions in men with more advanced disease remains to be determined. Second, diet and physical activity were assessed using self-reporting, and the instruments used may not have been sufficiently discriminative to detect small changes in lifestyle behaviors. Third, we did not include the participants’ caregivers or family/friends in the intervention. In our past trial of a structured partnered exercise program for prostate cancer patients and their spouses, retention and adherence rates to exercise in patients exceeded that in our other patient-only trials [9]. Therefore, partner support may be a key facilitator for patient behavior change. Improving the health of partners may also have a positive impact on patients’ health, a concept we are currently testing in a clinical exercise trial for cancer survivors and their spouse (or partners; NCT03630354). Fourth, we selected one type of physical activity tracker for integration with the web portal, and several comments made in the exit survey indicated that people would prefer more options and to use the devices they already owned (see Multimedia Appendix 2 for participant feedback). Participant feedback also indicated that additional programming to personalize and update the recommendations and messaging over time was desired. Accordingly, our team is currently enrolling individuals going to surgery for prostate cancer (Prostate 8-II trial, NCT #03999151) to a longer intervention (24 months) with more tailored feedback that adjusts to real-time self-reported diet and exercise data. P8-II also provides quarterly health coaching. Finally, it is worth noting that nonserious study-related AEs increased across intervention levels. However, the AEs reported were consistent with expected side effects of increased physical activity (eg, muscle pain, fatigue) and/or dietary change (eg, fatigue) and all were mild to moderate.

### Conclusions

The TrueNTH Community of Wellness trial demonstrated the feasibility of a web-based, remotely delivered, tailored behavioral intervention among individuals with all stages of prostate cancer. Men in level 4 who received two 30-minute phone calls reported higher satisfaction, engaged more frequently with the intervention, and reported small improvements in diet and physical activity compared with men in level 1. Future studies are warranted to evaluate how to increase the effect of the intervention on lifestyle behaviors, while maintaining long-term sustainability and scalability, as well as designing and implementing behavioral interventions for more diverse populations.

## Appendix

Multimedia Appendix 1 The components and points assigned for the lifestyle behavior score.

Multimedia Appendix 2 Participant feedback from a 3-month technology-supported behavioral intervention for prostate cancer survivors.

Multimedia Appendix 3 Baseline, 3-month, 6-month, and change from baseline lifestyle, diet, and physical activity scores among men with prostate cancer participating in a 3-month technology-supported behavioral intervention, randomized by level.

Multimedia Appendix 4 Difference in mean change in an overall lifestyle score (range 0-20) from baseline to 6 months, comparing intervention levels 2 to 4 with level 1. Higher scores indicate more healthy lifestyle behaviors.

Multimedia Appendix 5 Differences in mean change in an overall diet score (range 0-14) and servings per day of diet items from baseline to 6 months, comparing intervention levels 2 to 4 with level 1. A higher score for the overall diet score indicates healthier diet behaviors. The intervention aimed to increase intake of cruciferous vegetables, fish, tomatoes, and vegetable fat and decrease intake of poultry with skin, processed meat, and whole milk.

Multimedia Appendix 6 Difference in mean change in an overall physical activity score (range 0-6) and types of physical activity (minutes per week of aerobic; sessions per week of strength training and stretching) from baseline to 6 months, comparing intervention levels 2 to 4 with level 1. Higher scores indicate more physical activity.

Multimedia Appendix 7 CONSORT-eHEALTH checklist (V1.6.1).

## Abbreviations

AE: adverse event
CHAMPS: Community Health Activities Model Program for Seniors
FFQ: food frequency questionnaire
OHSU: Oregon Health and Sciences University
NIH: National Institutes of Health
P8-I: Prostate 8-I
UCD: University of Colorado Denver
UCSF: University of California San Francisco
