# Supplementary material for: Feasibility and Acceptability of a Remotely Delivered, Web-Based Behavioral Intervention for Men With Prostate Cancer: Four-Arm Randomized Controlled Pilot Trial
Source: J Med Internet Res. 2020 Dec 31;22(12):e19238. doi: 10.2196/19238 (PMC7808895; doi:10.2196/19238)
Supplement: Multimedia Appendix 1 [file jmir_v22i12e19238_app1.docx]

| **Supplementary Table 1.** Components and points assigned for a composite lifestyle behavior score. Lifestyle behaviors included were selected from the literature as factors associated with risk of prostate cancer recurrence or mortality. | | | | |
| --- | --- | --- | --- | --- |
| **Food category** | | **Cutpoints, serv/week** | **Point value assigned** | |
| **Whole milk** | | >1 | 0 | |
|  |  | >0 to 1 | 1 | |
|  |  | none | 2 | |
| **Poultry with skin** | | >1 | 0 | |
|  |  | >0 to 1 | 1 | |
|  |  | none | 2 | |
| **Processed meat** | | >1 | 0 | |
|  |  | >0 to 1 | 1 | |
|  |  | none | 2 | |
| **Tomatoes** | | < 1 | 0 | |
|  |  | 1 to <2 | 1 | |
|  |  | ≥2 | 2 | |
| **Fish** | | < 1 | 0 | |
|  |  | 1 to <2 | 1 | |
|  |  | ≥2 | 2 | |
| **Cruciferous vegetables** | | <5 | 0 | |
|  |  | 5 to <7 | 1 | |
|  |  | ≥7 | 2 | |
| **Vegetable fat** | | <5 | 0 | |
|  |  | 5 to <7 | 1 | |
|  |  | ≥7 | 2 | |
| **Physical activity** | | **Cutpoints** |  | |
| **Moderate to vigorous aerobic activity** | | <90 min/wk | 0 | |
|  |  | 90 to <150 min/wk | 1 | |
|  |  | ≥150 min/wk | 2 | |
| **Strength training** | | none | 0 | |
|  |  | 1 session/wk OR multiple sessions/wk totaling <60 minutes | 1 | |
|  |  | ≥2 sessions/wk and ≥60 minutes/wk | 2 | |
| **Stretching/flexibility** | | <2 sessions/wk | 0 | |
|  |  | 2 to <5 sessions/wk | 1 | |
|  |  | ≥5 sessions/wk | 2 | |
| **Total score, possible range:** |  | | | 0 - 20 |
