# Supplementary material for: Feasibility and Acceptability of a Remotely Delivered, Web-Based Behavioral Intervention for Men With Prostate Cancer: Four-Arm Randomized Controlled Pilot Trial
Source: J Med Internet Res. 2020 Dec 31;22(12):e19238. doi: 10.2196/19238 (PMC7808895; doi:10.2196/19238)
Supplement: Multimedia Appendix 2 [file jmir_v22i12e19238_app2.docx]

| Supplementary Table 2. Participant feedback from a 3-month technology-supported behavioral intervention for prostate cancer survivors. |
| --- |
|  |
| *On-boarding process and general impression of portal* |
| “Surveys are too long. Don't have time for them.” (Level 2) |
| “Things were difficult to understand and navigate.” (Level 1) |
| Security requirements were too onerous to continue. (Level 1) |
| “Overall it does not appear to have eye-catching appeal. (Picky, I know). Pages froze up on me. Log-in was rejected. Error messages were received.” (Level 2) |
|  |
| *Format* |
| “Intervention was generally stuff I was already doing, but is a good compendium. It would be more useful to me if it were an app that logged progress, set reminders, etc., from phone. See above: I like an app. Similar in concept to a fitness tracking app or MyFitnessPal for logging calories and excer.” (Level 2) |
|  |
| *Time commitment* |
| “I work full time and did not have time to devote to the program.” (Level 3) |
|  |
| *Interactive features* |
| “I recently read about HIIT workouts for seniors that would be good to add. Some ability to take tests or be more interactive to see that you get and retain what is being communicated, to sort of drill it into your consciousness. Kind of like the Eat This or Eat That quizzes.” (Level 1) |
| “I didn't feel a need to visit the portal unless I was completing some kind of survey.” (Level 3) |
| “More of an interaction and asking more questions of what we are currently doing. I feel as if the group has quite a wide expanse of what we already do. For example I tend to ride bikes about 50 to 100 miles a week and I imagine there are others that exercise quite a bit too.” (Level 3) |
| “I enjoyed the study. The thing that was the most helpful to me were the prescriptions and the accountability of having to report every week.” (Level 4) |
|  |
| *Feedback on progress* |
| “Need coaching and feedback from peers” (Level 2) |
| “Out of sight, out of mind. Not being used much, too many other good options appear to me first. I need reminders to continue my prescribed plan!” (Level 2) |
| “Maybe weekly summary of exercise in specific time/distant at individual activities.” (Level 2) |
| “The weekly progress reports should be sent on a Sunday with instructions that it covers the previous week, Sunday to Saturday.” (Level 3) |
| “I'd like to be able to see the data from the full 12 weeks.. Also when it started I wasn't aware that I would need to go into the portal on the same day each week to update it. If I missed a day, I think you lost that data.. so I wish there was maybe a 10-day sweep with reminders every 7 days.” (Level 4) |
| “have a scheduled discussion monthly with the coach instead of just once, in the middle of the study.” (Level 4) |
| “Offer follow up interviews and/or email communication with Diet Coach and Exercise Coach. One-half hour to one hour telephone interview was an excellent opportunity to ask specific questions and seek advise and direction for the Community of Wellness research program, and was a terrific motivator. Scheduling a time certain follow up personal communication and/or email follow up on a certain time schedule might engender better compliance with the program.” (Level 4) |
| “As a man I LIKE TO SEE RESULTS of extensive efforts. I and many others, I assume, spent considerable time with all of these questionnaires: always "just one or two more" we were told, and then after the program ended there was no measurement to see where our efforts had any effect; i.e. did life style changes occur; did PSA stay steady, go down, or up; was there correlation between certain foods or habits that influenced PSA; any changes indicated in morbidity??? Since we didn't know where we were going with the study and we answered your questions, it would have been MUCH more motivational if we could ask questions, get feedback and direction, and have a measurable goal or desired result…” (Level 4) |
|  |
| *Content suggestions* |
| “Specific meal plans. Most of what I saw seemed the same as what I could see on many other dietary health web sites. It didn't seem to include unique info. Making it unique as well as meal plan helpful might help.” (Level 1) |
| “I read the information initially and that was enough. There were no reasons to go back. This information would probably be better provided as a short pamphlet - there was no advantage having it online.” (Level 1) |
| “More healthy recipes for diet. All I have tried have been very good, and have added in the way I eat” (Level 2) |
| “Physical limitations could be considered i.e. inability to do sustained aerobic exercises, access to appropriate use equipment (expenses involved). Isolation in semi-rural area.” (Level 2) |
| “Weekly short membership notes about pertinent subjects like diet, nutrition, exercise--basically a keeping in contact and education element.” (Level 2) |
| “Information provided for the diet section I feel was somewhat incomplete. I tried to follow the requirements for the cruciferous vegetables but didn't really understand why. I may have been more diligent had more been explained as to how these vegetables are important in fighting cancer.” (Level 2) |
| “Didn't understand why cooked tomatoes was on diet and not fresh. Supplements are needed if well balanced diet isn't possible” (Level 2) |
| “Lack of understanding on my part.” (Level 2) |
| “There is no differentiation between upper body and lower body such that one could do 20 minutes of upper and 10 minutes of lower. Is one more important or be given more attention to? In addition, I did not really care for the yoga experience and tried tai chi at the end of the study which I cared for only somewhat more.” (Level 2) |
| “Intervention was generally stuff I was already doing, but is a good compendium. It would be more useful to me if it were an app that logged progress, set reminders, etc., from phone. See above: I like an app. Similar in concept to a fitness tracking app or MyFitnessPal for logging calories and excer.” (Level 2) |
| “A set of ideas about how to stay on plan while traveling and eating out for an extended period. If one is still working it is harder to follow the plan.” (Level 3) |
| “More reporting re study findings vis a vis the recommended lifestyle changes and how same correlate to survival/remission data.” (Level 3) |
| “If periodic articles or updates on current research focusing on prostate cancer were available on the portal then I missed them. If that was not part of the site then this would be a helpful addition. Perhaps combined with links to that kind of material from sources you trust.” (Level 3) |
| “Overall my only "issue" was it was information overload. I already track my fitness with my Garmin device, Rx plan and Healthy Habits on my employer's insurer's website. I think the idea of a comprehensive "health dashboard" is fantastic but I didn't invest the time to really capitalize on your portal.” (Level 3) |
| “Information is too generic. I am not a fan of spending time looking at information on the web.” (Level 4) |
| “more explicit exercise information and diagrams” (Level 4) |
| “The issue with the diet prescription was that it was too narrow and really didn't provide feedback. It was static and should have been dynamic.” (Level 4) |
| “I would love to see a site that provided a variable weekly plan for the exercise prescription. Basically an exercise plan generator where, perhaps, you select one or more top level categories (e.g., Resistance, Cardio, Flexibility) and then click a button to generate a new plan for the selected categories. It is easy to get into an exercise rut where one repeats the same old exercises over and over again. Variability is a good thing!” (Level 4) |
|  |
| *Tailoring / personalization* |
| “The study was quite impersonal. I would prefer some voice contact during the study to discuss the results and comments from the staff.” (Level 1) |
| “Did not offer advice/help/assistance to those of us who are cancer free but still have side effects of our prostate cancer treatment.” (Level 1) |
| “I get maybe 100 messages each day, and the emails from TrueNTH look like random solicitations so I often miss them.” (Level 1) |
| “Don't know, but having a personal; on-line "coach" might make me more attentive to messages.” (Level 1) |
| “resources specific to the community or region where the participant lives” (Level 1) |
| “It seemed pretty generic. For all the questions we had to answer, I would have expected something more specific to me.” (Level 3) |
| “I've always been self-motivated with exercising so I didn't use any of the exercise prescription.” (Level 3) |
| “I didn't feel that I could fully express myself and detail my circumstances within the limited parameters of the site. I had several impediments to exercise during the period which could not be compensated for during the term of the program. I'm vegetarian/vegan and there didn't seem to be any consideration for such dietary choices.” Would have liked “more specific info based on feedback from the participant” (Level 4) |
|  |
| *Text messages* |
| “I am not in the habit of texting-so do not use the feature much. (HANDS too BIG)” (Level 3) |
| “As a friendly reminder it was nice but since it wasn't tied to any specific activity effecting me - I stopped reading them.” (Level 3) |
| “I didn't find the prompts necessary at all.” (Level 3) |
| “Only a few messages were relevant for me and I got to the point where the 10:00 a.m. MT text almost every day was annoying.” (Level 3) |
| “i do not use text messaging” (Level 4) |
| “The messages were good, but most of the things I was already doing.” (Level 4) |
|  |
| *Fitbit* |
| “[The Fitbit] was a motivator however, as I would sometimes go for an extra walk just to try to reach my 10,000 step daily goal.” (Level 3) |
| “I liked the Fitbit integration. Making that more robust would be great.” (Level 3) |
| “I never could link the daily monitoring device with the portal” (Level 3) |
| “[The fitbit] does not accurately measure activity. I thought it was useless.” (Level 3) |
