# Supplementary material for: Feasibility and Acceptability of a Remotely Delivered, Web-Based Behavioral Intervention for Men With Prostate Cancer: Four-Arm Randomized Controlled Pilot Trial
Source: J Med Internet Res. 2020 Dec 31;22(12):e19238. doi: 10.2196/19238 (PMC7808895; doi:10.2196/19238)
Supplement: Multimedia Appendix 3 [file jmir_v22i12e19238_app3.docx]

| **Supplementary Table 3.** Baseline, 3-month, 6-month, and change from baseline lifestyle, diet, and physical activity scores among men with prostate cancer participating in a 3-month technology-supported behavioral intervention, by level randomized.^a^ | | | | | | | | |
| --- | --- | --- | --- | --- | --- | --- | --- | --- |
|  | **Level 1** | | **Level 2** | | **Level 3** | | **Level 4** | |
| **Lifestyle score**^b^ | **N** | **Median (IQR)** | **N** | **Median (IQR)** | **N** | **Median (IQR)** | **N** | **Median (IQR)** |
| Baseline | 48 | 7.0 (4.0, 9.0) | 48 | 6.0 (4.0, 8.0) | 50 | 7.0 (5.0, 9.0) | 51 | 7.0 (4.0, 9.0) |
| 3-Month | 36 | 8.0 (6.0, 9.0) | 37 | 7.0 (6.0, 9.0) | 36 | 8.0 (6.0, 9.0) | 37 | 9.0 (8.0, 10.0) |
| 6-Month | 35 | 7.0 (6.0, 9.0) | 36 | 7.0 (6.0, 8.0) | 34 | 8.0 (7.0, 10.0) | 36 | 8.0 (7.0, 10.0) |
| Change, baseline to 3 mo. | 36 | 1.0 (-1.0, 2.0) | 37 | 1.0 (0.0, 3.0) | 36 | 0.0 (-1.0, 2.0) | 37 | 2.0 (0.0, 3.0) |
| Change, baseline to 6 mo. | 35 | 0.0 (-1.0, 2.0) | 36 | 1.0 (-1.0, 2.5) | 34 | 0.5 (-1.0, 2.0) | 36 | 1.0 (0.0, 3.0) |
| **Diet score**^c^ |  |  |  |  |  |  |  |  |
| Baseline | 48 | 5.0 (4.0, 5.0) | 48 | 4.0 (4.0, 5.0) | 50 | 5.0 (4.0, 5.0) | 51 | 4.0 (4.0, 5.0) |
| 3-Month | 36 | 5.0 (4.0, 5.0) | 37 | 5.0 (4.0, 5.0) | 36 | 5.0 (4.0, 5.0) | 37 | 5.0 (5.0, 6.0) |
| 6-Month | 35 | 5.0 (4.0, 5.0) | 36 | 4.0 (4.0, 5.0) | 34 | 4.0 (4.0, 6.0) | 36 | 5.0 (4.0, 6.0) |
| Change, baseline to 3 mo. | 36 | 0.0 (0.0, 1.0) | 37 | 0.0 (0.0, 1.0) | 36 | 0.0 (0.0, 1.0) | 37 | 1.0 (0.0, 2.0) |
| Change, baseline to 6 mo. | 35 | 0.0 (0.0, 1.0) | 36 | 0.0 (0.0, 1.0) | 34 | 0.0 (-1.0, 0.0) | 36 | 1.0 (0.0, 1.0) |
| **Cruciferous vegetables, serv./d** |  |  |  |  |  |  |  |  |
| Baseline | 48 | 0.3 (0.1, 0.7) | 48 | 0.4 (0.2, 0.8) | 50 | 0.4 (0.1, 0.9) | 51 | 0.3 (0.1, 0.7) |
| 3-Month | 36 | 0.4 (0.3, 0.9) | 37 | 0.6 (0.3, 1.0) | 36 | 0.8 (0.4, 1.3) | 37 | 1.0 (0.5, 1.6) |
| 6-Month | 35 | 0.4 (0.3, 1.1) | 36 | 0.6 (0.2, 1.1) | 34 | 0.8 (0.4, 1.4) | 36 | 0.8 (0.5, 1.4) |
| Change, baseline to 3 mo. | 36 | 0.1 (-0.1, 0.3) | 37 | 0.1 (0.0, 0.4) | 36 | 0.3 (0.1, 0.7) | 37 | 0.5 (0.2, 0.9) |
| Change, baseline to 6 mo. | 35 | 0.1 (0.0, 0.3) | 36 | 0.1 (0.0, 0.3) | 34 | 0.4 (0.2, 0.6) | 36 | 0.4 (0.2, 0.6) |
| **Tomatoes, serv./d** |  |  |  |  |  |  |  |  |
| Baseline | 48 | 0.3 (0.1, 0.5) | 48 | 0.3 (0.1, 0.4) | 50 | 0.3 (0.1, 0.5) | 51 | 0.2 (0.1, 0.5) |
| 3-Month | 36 | 0.3 (0.2, 0.5) | 37 | 0.4 (0.2, 0.6) | 36 | 0.3 (0.1, 0.6) | 37 | 0.4 (0.2, 0.7) |
| 6-Month | 35 | 0.3 (0.2, 0.6) | 36 | 0.3 (0.2, 0.5) | 34 | 0.3 (0.1, 0.7) | 36 | 0.3 (0.2, 0.6) |
| Change, baseline to 3 mo. | 36 | 0.0 (-0.1, 0.1) | 37 | 0.1 (-0.1, 0.2) | 36 | 0.1 (-0.1, 0.2) | 37 | 0.1 (-0.1, 0.3) |
| Change, baseline to 6 mo. | 35 | -0.1 (-0.1, 0.2) | 36 | 0.1 (-0.1, 0.1) | 34 | 0.1 (-0.1, 0.2) | 36 | 0.1 (-0.1, 0.2) |
| **Fish, serv./d** |  |  |  |  |  |  |  |  |
| Baseline | 48 | 0.2 (0.1, 0.3) | 48 | 0.1 (0.0, 0.3) | 50 | 0.2 (0.0, 0.4) | 51 | 0.1 (0.0, 0.2) |
| 3-Month | 36 | 0.2 (0.1, 0.4) | 37 | 0.2 (0.1, 0.3) | 36 | 0.3 (0.2, 0.5) | 37 | 0.3 (0.1, 0.6) |
| 6-Month | 35 | 0.3 (0.1, 0.4) | 36 | 0.2 (0.1, 0.3) | 34 | 0.3 (0.1, 0.4) | 36 | 0.2 (0.1, 0.5) |
| Change, baseline to 3 mo. | 36 | 0.0 (-0.1, 0.1) | 37 | 0.0 (-0.1, 0.1) | 36 | 0.1 (0.0, 0.2) | 37 | 0.1 (0.0, 0.3) |
| Change, baseline to 6 mo. | 35 | 0.0 (-0.1, 0.1) | 36 | 0.0 (-0.1, 0.1) | 34 | 0.1 (0.0, 0.1) | 36 | 0.1 (0.0, 0.1) |
| **Vegetable fat, serv./d** |  |  |  |  |  |  |  |  |
| Baseline | 48 | 1.2 (0.7, 1.9) | 48 | 1.1 (0.7, 1.8) | 50 | 1.4 (0.7, 2.5) | 51 | 1.2 (0.5, 1.6) |
| 3-Month | 36 | 1.4 (0.9, 2.6) | 37 | 1.0 (0.7, 2.2) | 36 | 1.9 (1.0, 3.1) | 37 | 1.6 (1.0, 2.4) |
| 6-Month | 35 | 1.5 (0.7, 2.3) | 36 | 1.1 (0.5, 1.9) | 34 | 2.1 (1.1, 3.2) | 36 | 1.5 (0.8, 2.2) |
| Change, baseline to 3 mo. | 36 | 0.1 (-0.3, 1.0) | 37 | 0.0 (-0.4, 0.4) | 36 | 0.2 (-0.6, 0.9) | 37 | 0.3 (-0.1, 0.8) |
| Change, baseline to 6 mo. | 35 | -0.1 (-0.5, 1.0) | 36 | 0.1 (-0.6, 0.6) | 34 | 0.0 (-0.4, 0.9) | 36 | 0.2 (0.0, 0.5) |
| **Poultry with skin, serv./d** |  |  |  |  |  |  |  |  |
| Baseline | 48 | 0.1 (0.0, 0.1) | 48 | 0.1 (0.0, 0.1) | 50 | 0.0 (0.0, 0.1) | 51 | 0.1 (0.0, 0.1) |
| 3-Month | 36 | 0.0 (0.0, 0.1) | 37 | 0.0 (0.0, 0.1) | 36 | 0.0 (0.0, 0.1) | 37 | 0.0 (0.0, 0.1) |
| 6-Month | 35 | 0.1 (0.0, 0.1) | 36 | 0.0 (0.0, 0.1) | 34 | 0.0 (0.0, 0.1) | 36 | 0.0 (0.0, 0.1) |
| Change, baseline to 3 mo. | 36 | 0.0 (0.0, 0.0) | 37 | 0.0 (-0.1, 0.0) | 36 | 0.0 (0.0, 0.0) | 37 | 0.0 (-0.1, 0.0) |
| Change, baseline to 6 mo. | 35 | 0.0 (0.0, 0.1) | 36 | 0.0 (-0.1, 0.0) | 34 | 0.0 (-0.1, 0.0) | 36 | 0.0 (-0.1, 0.0) |
| **Processed meat, serv./d** |  |  |  |  |  |  |  |  |
| Baseline | 48 | 0.2 (0.0, 0.4) | 48 | 0.3 (0.0, 0.7) | 50 | 0.2 (0.0, 0.4) | 51 | 0.3 (0.0, 0.7) |
| 3-Month | 36 | 0.1 (0.0, 0.3) | 37 | 0.2 (0.1, 0.4) | 36 | 0.1 (0.0, 0.3) | 37 | 0.1 (0.0, 0.3) |
| 6-Month | 35 | 0.1 (0.1, 0.3) | 36 | 0.2 (0.1, 0.4) | 34 | 0.1 (0.0, 0.3) | 36 | 0.1 (0.0, 0.3) |
| Change, baseline to 3 mo. | 36 | -0.1 (-0.2, 0.0) | 37 | -0.1 (-0.3, 0.1) | 36 | -0.1 (-0.3, 0.0) | 37 | -0.1 (-0.5, 0.0) |
| Change, baseline to 6 mo. | 35 | -0.1 (-0.2, 0.0) | 36 | -0.1 (-0.3, 0.1) | 34 | -0.1 (-0.3, 0.0) | 36 | -0.1 (-0.5, 0.0) |
| **Whole milk, serv./d** |  |  |  |  |  |  |  |  |
| Baseline | 48 | 0.0 (0.0, 0.0) | 48 | 0.0 (0.0, 0.0) | 50 | 0.0 (0.0, 0.1) | 51 | 0.0 (0.0, 0.0) |
| 3-Month | 36 | 0.0 (0.0, 0.0) | 37 | 0.0 (0.0, 0.0) | 36 | 0.0 (0.0, 0.0) | 37 | 0.0 (0.0, 0.0) |
| 6-Month | 35 | 0.0 (0.0, 0.0) | 36 | 0.0 (0.0, 0.0) | 34 | 0.0 (0.0, 0.1) | 36 | 0.0 (0.0, 0.0) |
| Change, baseline to 3 mo. | 36 | 0.0 (0.0, 0.0) | 37 | 0.0 (0.0, 0.0) | 36 | 0.0 (-0.1, 0.0) | 37 | 0.0 (0.0, 0.0) |
| Change, baseline to 6 mo. | 35 | 0.0 (0.0, 0.0) | 36 | 0.0 (0.0, 0.0) | 34 | 0.0 (0.0, 0.0) | 36 | 0.0 (0.0, 0.0) |
| **Physical activity score**^d^ |  |  |  |  |  |  |  |  |
| Baseline | 49 | 3.0 (1.0, 4.0) | 49 | 2.0 (1.0, 4.0) | 50 | 2.0 (1.0, 4.0) | 51 | 3.0 (1.0, 4.0) |
| 3-Month | 36 | 3.0 (2.0, 4.0) | 39 | 3.0 (2.0, 4.0) | 40 | 2.0 (2.0, 4.0) | 38 | 4.0 (3.0, 5.0) |
| 6-Month | 36 | 3.0 (1.5, 4.0) | 36 | 3.0 (1.0, 4.0) | 36 | 3.0 (2.0, 4.5) | 37 | 3.0 (2.0, 5.0) |
| Change, baseline to 3 mo. | 36 | 0.0 (-1.0, 1.0) | 38 | 0.0 (-1.0, 2.0) | 40 | 0.0 (-1.5, 1.0) | 38 | 1.0 (0.0, 2.0) |
| Change, baseline to 6 mo. | 36 | 0.0 (-1.0, 1.0) | 36 | 0.0 (-1.0, 1.0) | 36 | 0.0 (-1.0, 1.0) | 37 | 0.0 (0.0, 1.0) |
| **Moderate-to-vigorous physical activity, min./wk.** |  |  |  |  |  |  |  |  |
| Baseline | 49 | 210 (60, 360) | 49 | 105 (30, 270) | 50 | 233 (30, 435) | 51 | 225 (60, 450) |
| 3-Month | 36 | 210 (60, 398) | 39 | 165 (30, 345) | 40 | 210 (105, 330) | 38 | 255 (135, 450) |
| 6-Month | 36 | 218 (98, 458) | 36 | 180 (60, 353) | 36 | 255 (135, 450) | 37 | 315 (105, 375) |
| Change, baseline to 3 mo. | 36 | 0 (-120, 105) | 38 | 30 (-30, 135) | 40 | 0 (-203, 90) | 38 | 30 (-90, 150) |
| Change, baseline to 6 mo. | 36 | 15 (-83, 120) | 36 | 0 (-38, 195) | 36 | 0 (-83, 98) | 37 | 0 (-195, 120) |
| **Stretching, sessions/wk.** |  |  |  |  |  |  |  |  |
| Baseline | 49 | 0.0 (0.0, 4.0) | 49 | 2.0 (0.0, 4.0) | 50 | 1.0 (0.0, 3.0) | 51 | 1.0 (0.0, 3.0) |
| 3-Month | 36 | 2.0 (0.0, 3.5) | 39 | 3.0 (0.0, 5.0) | 40 | 2.0 (0.0, 3.5) | 38 | 2.0 (0.0, 4.0) |
| 6-Month | 36 | 1.0 (0.0, 4.0) | 36 | 3.0 (0.0, 5.0) | 36 | 2.0 (0.0, 3.5) | 37 | 2.0 (0.0, 4.0) |
| Change, baseline to 3 mo. | 36 | 0.0 (-1.5, 0.0) | 38 | 0.0 (0.0, 1.0) | 40 | 0.0 (-0.5, 1.5) | 38 | 0.0 (0.0, 2.0) |
| Change, baseline to 6 mo. | 36 | 0.0 (-0.5, 0.0) | 36 | 0.0 (0.0, 0.0) | 36 | 0.0 (-0.5, 1.0) | 37 | 0.0 (0.0, 2.0) |
| **Strength training, sessions/wk.** |  |  |  |  |  |  |  |  |
| Baseline | 49 | 0.0 (0.0, 3.0) | 49 | 0.0 (0.0, 2.0) | 50 | 0.5 (0.0, 3.0) | 51 | 2.0 (0.0, 3.0) |
| 3-Month | 36 | 0.0 (0.0, 3.0) | 39 | 1.0 (0.0, 3.0) | 40 | 1.0 (0.0, 3.0) | 38 | 3.0 (2.0, 3.0) |
| 6-Month | 36 | 1.0 (0.0, 3.0) | 36 | 0.0 (0.0, 3.0) | 36 | 2.0 (0.0, 3.0) | 37 | 2.0 (0.0, 3.0) |
| Change, baseline to 3 mo. | 36 | 0.0 (0.0, 0.0) | 38 | 0.0 (0.0, 1.0) | 40 | 0.0 (0.0, 1.0) | 38 | 0.0 (0.0, 1.0) |
| Change, baseline to 6 mo. | 36 | 0.0 (0.0, 0.0) | 36 | 0.0 (0.0, 0.5) | 36 | 0.0 (0.0, 0.5) | 37 | 0.0 (0.0, 0.0) |

^a^Total men included at baseline, 3- and 6- months may not equal 202 due to incomplete FFQ or CHAMPS questionnaires or losses to follow-up/consent withdrawals.

^b^The lifestyle score is an overall score of diet and exercise, ranging from is 0-20.

^c^The diet score ranges from 0-14, comprised of 2 possible points for each of the following: cruciferous vegetables, tomatoes, fish, vegetable fat, processed meat, poultry with skin, and whole milk intake.

^d^The exercise score ranges from 0-6, comprised of 2 possible points for each of the following: moderate to vigorous intensity aerobic physical activity, strength training, and stretching.
