# Supplementary figures and images for: Feasibility and Acceptability of a Remotely Delivered, Web-Based Behavioral Intervention for Men With Prostate Cancer: Four-Arm Randomized Controlled Pilot Trial
Source: J Med Internet Res. 2020 Dec 31;22(12):e19238. doi: 10.2196/19238 (PMC7808895; doi:10.2196/19238)

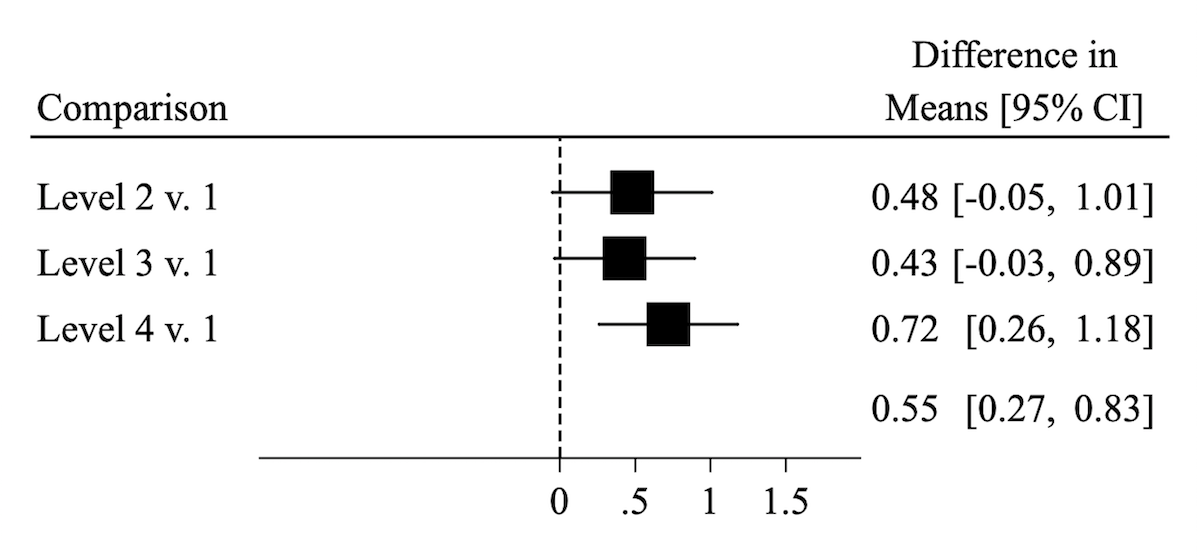

Supplement: Multimedia Appendix 4 [file jmir_v22i12e19238_app4.png]

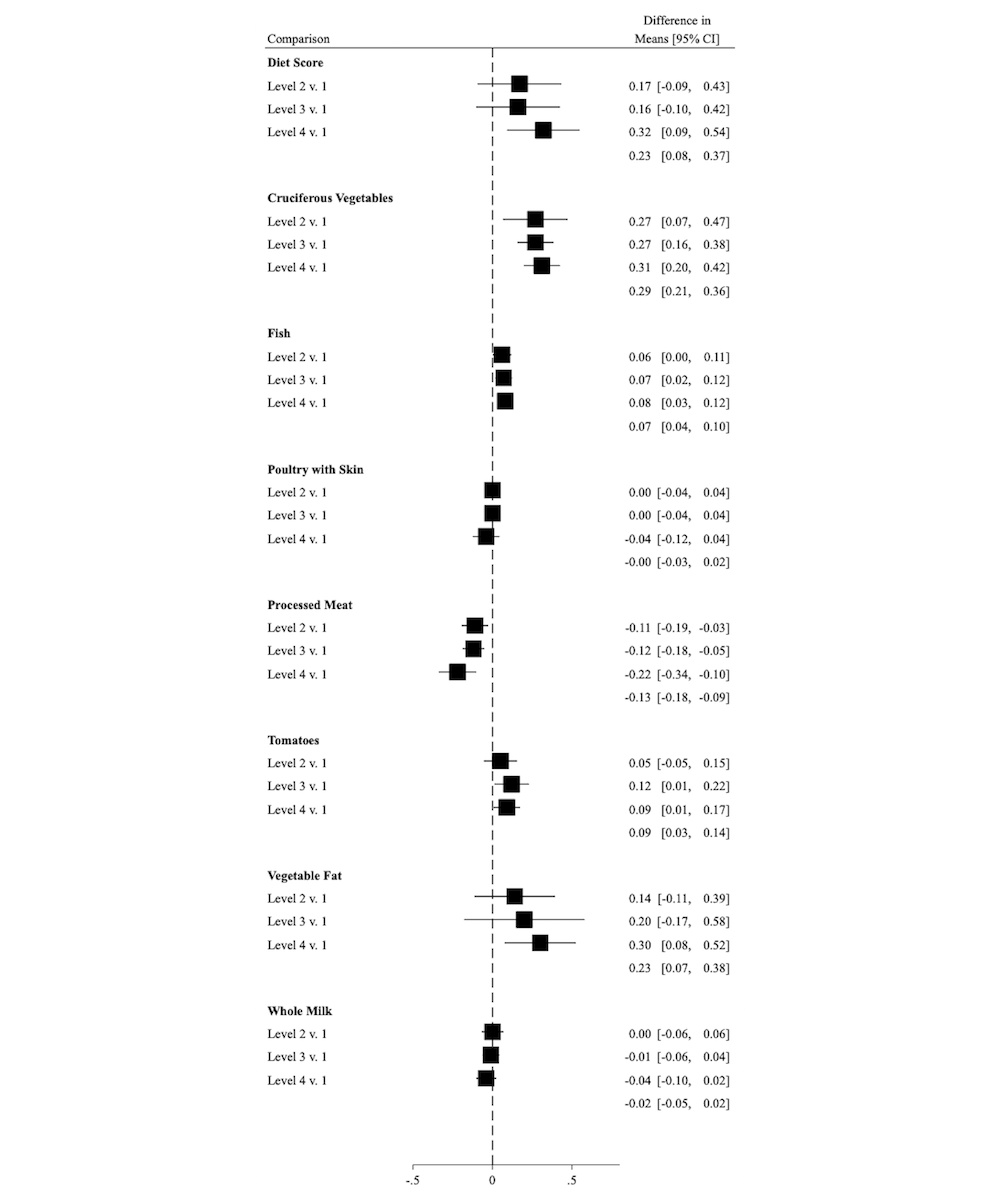

Supplement: Multimedia Appendix 5 [file jmir_v22i12e19238_app5.png]

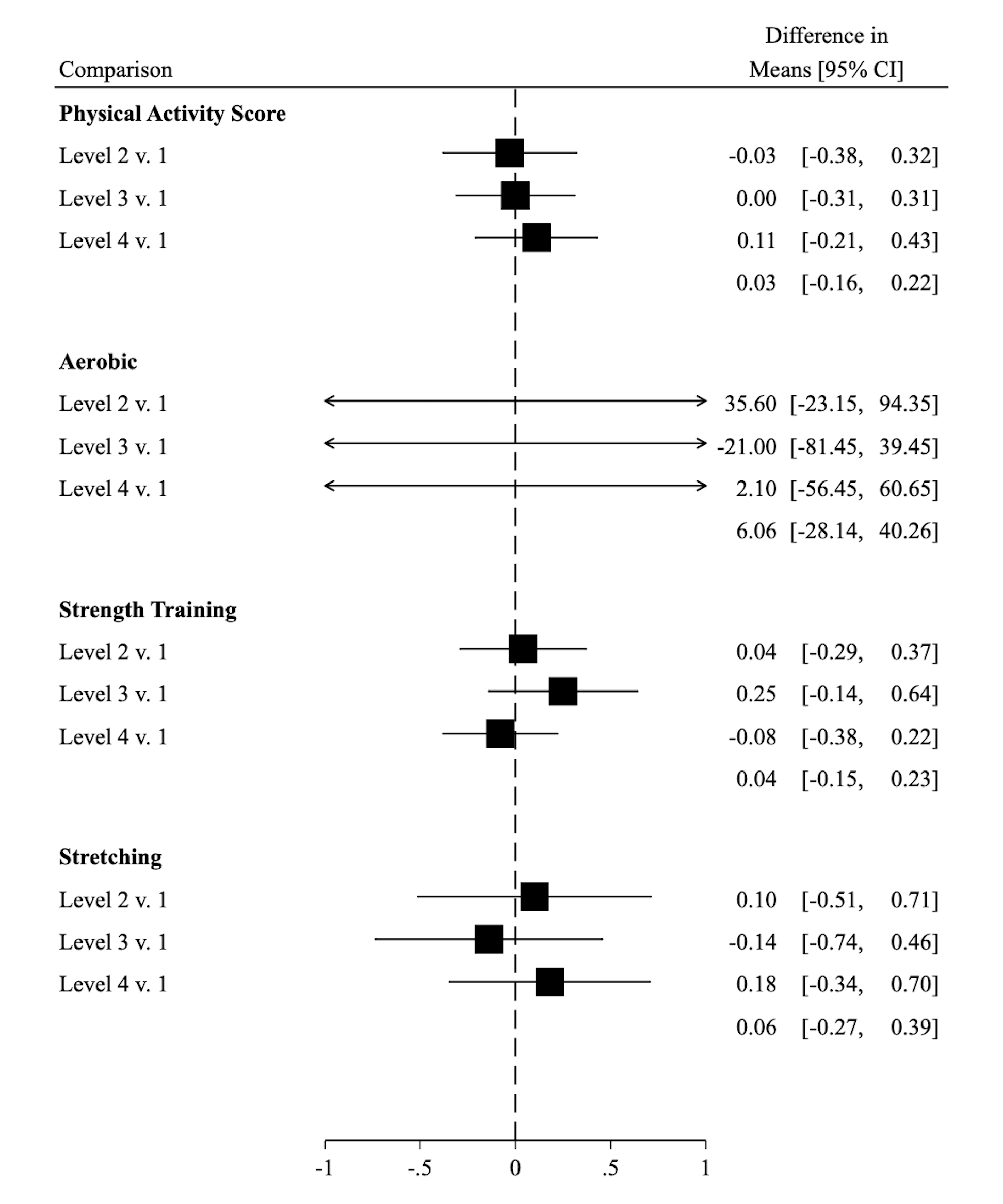

Supplement: Multimedia Appendix 6 [file jmir_v22i12e19238_app6.png]
